# Hall current effect in bioconvection Oldroyd-B nanofluid flow through a porous medium with Cattaneo-Christov heat and mass flux theory

**DOI:** 10.1038/s41598-022-23932-0

**Published:** 2022-11-17

**Authors:** Noor Saeed Khan, Somchai Sriyab, Attapol Kaewkhao, Ekkachai Thawinan

**Affiliations:** 1 Department of Mathematics, Division of Science and Technology, University of Education, Lahore, 54000 Pakistan; 2 Research Group in Mathematics and Applied Mathematics, Department of Mathematics, Faculty of Science, Chiang Mai University, Chiang Mai, 50200 Thailand

**Keywords:** Engineering, Mathematics and computing

## Abstract

Bioconvection due to microorganisms is important area of research, considerably importance for environment and sustainable fuel cell technologies. Buongiorno nanofluid model for Cattaneo-Christov heat and mass flux theory taken into account the Oldroyd-B nanofluid and gyrotactic microorganisms in a rotating system with the effects of Hall current, and Darcy porous medium is scrutinized. The constitutive equations of the problem are transformed into nondimensional equations with the help of similarity transformations. Homotopy analysis method is used to obtain the solution. Graphs and table support the comprehesive representation of the achieved results. Radial velocity is reduced with the increasing values of relaxation time, retardation time and magnetic field parameters while heat transfer is augmented with thermal relaxation time parameter. The nanoparticles concentration is reduced with the increasing values of Schmidt number and the gyrotactic microorganisms concentration is enhanced with the increasing values of Peclet number. A nice agreement is obtained while comparing the present results numerically with the published results. The proposed mathematical model is used in biochemical engineering, meteorology, power and transportation production, optoelectronic and sensing microfabrication.

## Introduction

Heat transfer performance is enhanced on account of adding the micro size particles in the base liquids. The improved heating conduction of nanofluids has shown better results. Nanofluids are used in cooling of metalic plates, paper productions, drawing of plastic sheets, aerodynamics, etc. When the nanofluids are regulated by magnetic field, then these have potential applications such as anticancer drugs with the magnetic nanoparticles. Considering the applied magnetic field, Mandal and Pal^1^ analyzed the carbon nanotubes nanofluid with homogeneous-heterogeneous chemical reactions, variant heating transportation and mass diffusion, binary chemical reaction and activation energy using the convective boundary conditions. Yaseen et al.^2^ worked on the magnetohydrodynamic hybrid and mono nanoliquids within two shrinking and revolving discs through a Darcy-Forchheimer permeable medium considering Cattaneo-Christov (C-C) heat flux theory, viscous dissipation, Ohmic heating, solar radiation and heating generating/absorbing phenomena. Mandal^3^ used the fifth order Runge-Kutta-Fehlberg method in the presence of shooting procedure to solve the problem of convection boundary layer flow and heat transfer of micropolar nanofluid containing four types of nanoparticles on a nonlinear expanding source. Garia et al.^4^ scrutinized the magnetohydrodynamic motion of water based hybrid suspension on two surfaces by implementing the Das and Tiwari concept by considering the C-C heating transportation theory. They computed the coefficients of correlation of heating transportation and skin friction processed by the evaluation of probable error and statistical declaration. Pal and Mandal^5^ obtained the dual solutions of mixed convection-radiation stagnation point motion of three nanofluids through a porous medium over an expanding/contracting space by using the Runge-Kutta-Fehlberg procedure in connection to shooting algorithm which shows that the size of boundary layer is high for second solution compared to that of first solution. Rawat and Kumar^6^ worked on the water based copper nanofluid stagnation point flow with C-C concept and heating generating/absorbing phenomena, thermal radiation, activation energy, suction and slip condition which proved that thermal relaxation and radiation parameters enhance the heat transfer while slip and suction parameters reduce the mass transfer. Some nanofluids literature can be read from the references^7–16^.

Recently, non-Newtonian liquids have gained a wide range of importance in manufacturings, commerce and mechanics which are seen in food processsing, material handling, oil storage, warehousing etc. In general, non-Newtonian suspensions possess three leading classifications as differential, rate and integral types in which the rate type shows the stress relaxation. Oldroyd-B class holds the rate-type fluid which possess the generalization of the upper convected viscoelastic Maxwell fluid class in the possession of retardation time and presents the motion of viscoelastic fluids propposed by Oldroyd^17^. Hafeez et al.^18^ looked over the Oldroyd-B fluid in the existence of C-C heating transportation theory, homogeneous-heterogeneous reactions by using the BVP Midrich technique in which the flow speed becomes slow due to the effect of relaxation time factor. Shahzad et al.^19^ worked on the Das and Tiwari nanoliquid concept to investigate the thermal characteristics of Oldroyd-B fluid with engine oil as a conventional base suspension. They proved that the heating conduction of molybdenum disulfide engine oil nanofluid is greater than the copper engine oil nanofluid. Irfan et al.^20^ performed the analysis of double stratification in nonlinear radiative flow of Oldroyd-B nanofluid in stagnation region with the effects of magnetohydrodynamic and heat source/sink, Brownian diffusion and thermophoresis. Anwar et al.^21^ explained the transient free convection motion of an Oldroyd-B liquid through a vertical porous channel with nonlinear solar radiation in which the one side flow direction has unsteady velocity, while other side performs no movement on the basis of momentum conservation law and Fourier’s principle of heat transfer. Irfan et al.^22^ used the convective conditions in chemically reactive radiated Oldroyd-B nanofluid with buoyancy, magnetic and Joule heating effects. The different chrateristics of Oldroyd-B nanofluid are investigated by Irfan et al.^24–26^. Khan et al.^23^ investigated the Oldroyd-B liquid taken into account the C-C heat and mass flux theory using Darcy-Forchheimer effect to probe the bioconvection heating and nanoparticles concentration retaining gyrotactic microbs through a permeable source. Hafeez et al.^27^ worked on the modelling of heating transportation and nanoparticles concentration in the dynamics of Oldroyd-B nano-suspension with C-C heat and mass flux theory taken into account the Buongiorno nanofluid formulation. Usman et al.^28^ focused on the behavior associated with Oldroyd-B nanofluid thin film sprinkled on an extended cylinder accommodating nanoparticles and gyrotactic microorganisms with heat transfer by implementing the Homotopy Analysis Method (HAM). Khan et al.^29^ considered the Oldroyd-B fluid configured by infinite stretching disks with slip effects and homogeneous-heterogeneous chemical reactions. Hashmi et al.^30^ formulated a problem discussing the heat source/sink, viscous dissipation and Joule heating in mixed convection axisymmetric flow of an incompressible, electrically conducted Oldroyd-B fluid in two infinite isothermal stretching disks. Tong et al.^31^ presented the thermal behaviors of bioconvection Oldroyd-B nanofluid in the light of slip, thermal radiation, activation energy, magnetic force and porous medium effects with Cattaneo-Christov heat and mass flux theory. Hashmi et al.^32^ considered the steady heating and mass transferring motion of Oldroyd-B fluid with isothermal/exothermic reaction incarporating the features of Ohmic dissipation and Joule heating. The non-Newtonian features and others properties of various suspensions are exist in the studies^33–38^.

Bioconvection takes place in the suspension on account of up-swimming microorganisms which leads to the unstable density stratification, shows considerably importance for environment and sustainable fuel cell technologies having uses in environmental systems like ocean algae, fuel cells and biological polymer synthesis. The addition of nanofluid and bioconvection has obtained very important achievements in micro-fluidic devices including micro-reactors and micro-channel. Waqas et al.^39^ addressed the bioconvection effect due to microorganisms in Jeffery nanofluid past an expanding surface by considering the magnetic dipole effect. Raja et al.^40^ applied the soft computing based backpropagated neural networks with Levenberg marquardt technique to a bioconvection second-grade nanofluid model with C-C heat and mass flux theory, Hall current effect and porous medium. Waqas et al.^41^ discussed the numerical study of Oldroyd-B nanofluid flow with heat and mass transfer, gyrotactic microorganisms past a rotating disk through bvp4c built-in function of MATLAB software. Khan et al.^42^ analyzed the bioconvection nano-suspension motion and entropy generation in rotating system with the application of Homotopy Analysis Method. Rashad and Nabwey^43^ described the bioconvection of a nanofluid pertaining to motile microorganisms over a horizontal circular cylinder under the convective boundary conditions using Buongiorno’s nanofluid model with the Oberbeck-Boussinesq approximation. Alwatban et al.^44^ analyzed the bioconvection in magnetic nanoparticles using Wu’s slip effects near the surface. Lv et al.^45^ introduced a new form of non-Newtonian liquid called Reiner-Rivlin nanofluid past a rotating disk with C-C heat flux through a porous media in the presence of gyrotactic microorganisms. Shehzad et al.^46^ studied the bioconvection flow of Maxwell fluid past the isolated disk by considering the Buongiorno nanofluid model with Cattaneo-Christov energy and mass species flux models. Related studies are mentioned in the references^47–51^.

The aforementioned literature contains different aspects associated with different fluids and are interesting studies. Strong applied magnetic field with permeable media associated to gyrotactic microorganisms along a revolving disk in the presence of Oldroyd-B nano-suspension needs attention to be investigated. So the authors studied the Oldroyd-B nano-suspension motion over an expandable revolving disc in the light of C-C model soved through^52^.

## Procedure

### Problem formulation

**Figure 1:**
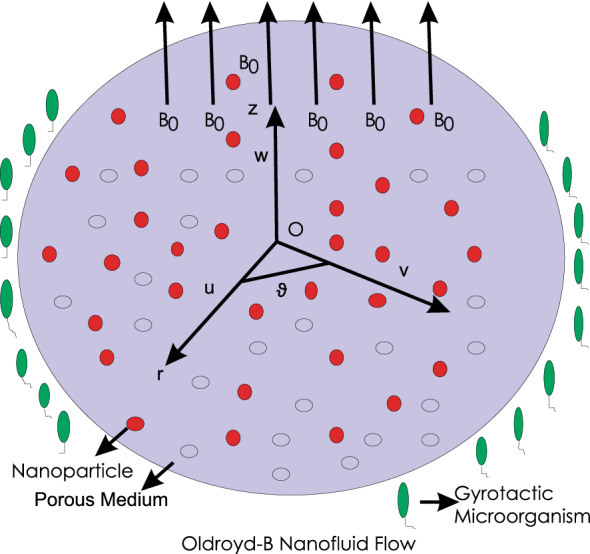
A plot presents the modeling.

The swirl of Oldroyd-B nanofluid retaining gyrotactic microorganisms in porous space for three dimensions is modelled. Heat and mass transfer modeling is obtained through C-C concept. For Hall current effect, strong magnetic field is in implementation to perpendicular side (consider the Fig. 1).

Considered problem has the dynamic mechanisms^18,27,28,42^.

1 $$\begin{aligned}{}&\frac{\partial u}{\partial r} + \frac{u}{r} + \frac{\partial w}{\partial z} = 0, \end{aligned}$$

2 $$\begin{aligned}{}&\rho _{f}\begin{pmatrix} u \frac{\partial u}{\partial r} + w \frac{\partial u}{\partial z} - \frac{v^{2}}{r}\end{pmatrix}\nonumber \\&\quad = \mu _{f}\frac{\partial ^{2} u}{\partial z^{2}} + \lambda _{1}\begin{pmatrix}\frac{uv^{2}}{r^{2}} + \frac{u^{2}\partial ^{2} u}{\partial r^{2}} + w^{2}\frac{\partial ^{2} u}{\partial z^{2}} + 2uw\frac{\partial ^{2} u}{\partial r \partial z} - \frac{2}{r}uv\frac{\partial v}{\partial r} - \frac{2}{r}vw\frac{\partial v}{\partial z} + \frac{1}{r^{2}}uv^{2} + \frac{1}{r}v^{2}\frac{\partial u}{\partial r}\end{pmatrix} \nonumber \\&\quad \quad - \lambda _{2} \mu _{f} \begin{bmatrix}-\frac{1}{r}\begin{pmatrix} \frac{\partial u}{\partial z}\end{pmatrix}^{2} - 2\frac{\partial u}{\partial z}\frac{\partial ^{2} w}{\partial z^{2}} + w \frac{\partial ^{3} u}{\partial z^{3}} - \frac{\partial u}{\partial r}\frac{\partial ^{2} u}{\partial z^{2}} - \frac{\partial u}{\partial z}\frac{\partial ^{2} u}{\partial r \partial z} + u \frac{\partial ^{3} u}{\partial r \partial z^{2}}\end{bmatrix} \nonumber \\&\quad \quad - \frac{\sigma _{f} \textit{B}^{2} _{0} (u - mv + w\lambda _{1} \frac{\partial u}{\partial z})}{1 + m^{2}}\nonumber \\&\quad \quad - \frac{\mu _{f}}{k}{} \textit{u} - k^{F}{u^{2}}, \end{aligned}$$

3 $$\begin{aligned}{}&\rho _{f}\begin{pmatrix} u \frac{\partial v}{\partial r} + w \frac{\partial v}{\partial z} + \frac{1}{r}uv\end{pmatrix} \nonumber \\&\quad = - \lambda _{1}\begin{pmatrix}-\frac{2vu^{2}}{r^{2}} + \frac{u^{2}\partial ^{2} v}{\partial r^{2}} + w^{2}\frac{\partial ^{2} v}{\partial z^{2}} + 2uw\frac{\partial ^{2} v}{\partial r \partial z} + \frac{2}{r}uv\frac{\partial u}{\partial r} + \frac{2}{r}vw\frac{\partial u}{\partial z} - \frac{2}{r^{2}}vu^{2} - \frac{1}{r^{2}}v^{3} + \frac{1}{r}v^{2}\frac{\partial v}{\partial r}\end{pmatrix}\nonumber \\&\quad \quad + \mu _{f}\frac{\partial ^{2} v}{\partial z^{2}} + \lambda _{2} \mu _{f} \begin{bmatrix}-\frac{u}{r}\frac{\partial ^{2} v}{\partial z^{2}}+ w \frac{\partial ^{3} v}{\partial z^{3}} - 2\frac{\partial v}{\partial z}\frac{\partial ^{2} w}{\partial z^{2}} - \frac{1}{r}\frac{\partial v}{\partial z}\frac{\partial u}{\partial z} + \frac{v}{r}\frac{\partial ^{2} u}{\partial z^{2}} - \frac{\partial ^{2} u}{\partial z^{2}}\frac{\partial v}{\partial r} - \frac{\partial v}{\partial z}\frac{\partial ^{2} u}{\partial r \partial z} - \frac{u}{r}\frac{\partial ^{2} v}{\partial z^{2}}\end{bmatrix} \nonumber \\&\quad \quad - \frac{\sigma _{f} {B^{2} _{0} }(v + mu + w\lambda _{1} \frac{\partial v}{\partial z})}{1 + m^{2}}\nonumber \\&\quad \quad - \frac{\mu _{f}}{k}{} \textit{w} - k^{F}{w^{2}}, \end{aligned}$$

4 $$\begin{aligned}{}&\begin{pmatrix} u \frac{\partial T}{\partial r} + w \frac{\partial T}{\partial z}\end{pmatrix} \nonumber \\&\quad = \alpha \frac{\partial ^{2} T}{\partial z^{2}} + 2\gamma _{1} \tau \frac{D_{T}}{T_{\infty }}\begin{bmatrix}u\frac{\partial T}{\partial z}\frac{\partial ^{2} T}{\partial r \partial z} + w \frac{\partial T}{\partial z}\frac{\partial ^{2} T}{\partial z^{2}}\end{bmatrix} + \tau \begin{bmatrix}D_{B}\begin{pmatrix}\frac{\partial T}{\partial z}\frac{\partial C}{\partial z}\end{pmatrix} + \frac{D_{T}}{T_{\infty }}\begin{pmatrix}\frac{\partial T}{\partial z}\end{pmatrix}^{2}\end{bmatrix}\nonumber \\&\quad \quad + \gamma _{1} \tau D_{B}\begin{bmatrix}u\frac{\partial T}{\partial z}\frac{\partial ^{2} C}{\partial r \partial z} + u\frac{\partial C}{\partial z}\frac{\partial ^{2} T}{\partial r \partial z} + w \frac{\partial T}{\partial z}\frac{\partial ^{2} C}{\partial z^{2}} + w \frac{\partial C}{\partial z}\frac{\partial ^{2} T}{\partial z^{2}}\end{bmatrix} \nonumber \\&\quad \quad +\gamma _{1}\begin{bmatrix}{} u \frac{\partial w}{\partial r}\frac{\partial T}{\partial z} + u \frac{\partial u}{\partial r}\frac{\partial T}{\partial r} + w \frac{\partial u}{\partial z}\frac{\partial T}{\partial r} + w \frac{\partial w}{\partial z}\frac{\partial T}{\partial z} + {u^{2}}\frac{\partial ^{2}T}{\partial r^{2}} + {w^{2}}\frac{\partial ^{2}T}{\partial z^{2}} + 2u {} w \frac{\partial ^{2}T}{\partial r \partial z}\end{bmatrix}, \end{aligned}$$

5 $$\begin{aligned}{}&u \frac{\partial C}{\partial r} + w \frac{\partial C}{\partial z} \nonumber \\&\quad = {D_{B}} \frac{\partial ^{2} C}{\partial z^{2}} + \gamma _{2} \tau \frac{D_{T}}{T_{\infty }}\begin{bmatrix}u\frac{\partial ^{3} T}{\partial r \partial z^{2}} + w \frac{\partial ^{3} T}{\partial z^{3}}\end{bmatrix} + \frac{D_{T}}{T_{\infty }}\frac{\partial ^{2} T}{\partial z^{2}}\nonumber \\&\quad \quad - \gamma _{2}\begin{bmatrix}{} u \frac{\partial w}{\partial r}\frac{\partial C}{\partial z} + u \frac{\partial u}{\partial r}\frac{\partial C}{\partial r} + w \frac{\partial u}{\partial z}\frac{\partial C}{\partial r} + w \frac{\partial w}{\partial z}\frac{\partial C}{\partial z} + {u^{2}}\frac{\partial ^{2}C}{\partial r^{2}} + {w^{2}}\frac{\partial ^{2}C}{\partial z^{2}} + 2u {} w \frac{\partial ^{2}C}{\partial r \partial z}\end{bmatrix}, \end{aligned}$$

6 $$\begin{aligned}{}&u \frac{\partial N}{\partial r} + w \frac{\partial N}{\partial z} + \frac{ bW_{c}}{(C_{w} - C_{\infty })}{\frac{\partial }{\partial z}\begin{pmatrix}{N}\frac{\partial C}{\partial z}\end{pmatrix}} = D_{n}\begin{bmatrix}\frac{\partial ^{2} N}{\partial r^{2}} + \frac{1}{r}\frac{\partial N}{\partial r} + \frac{\partial ^{2}N}{\partial z^{2}}\end{bmatrix}, \end{aligned}$$

using B.C. (boundary conditions)

7 $$\begin{aligned}{}&u = \textit{r}{} a ,\,\,{} v = \textit{r}\Omega ,\,\, w = 0, \,\, T = {T_{w}}, \,\,{} C = {C_{w}}, \,\,{} N = {N_{w}} \,\, at \,\, z = 0, \end{aligned}$$

8 $$\begin{aligned}{}&u \rightarrow {0},\,\,{} v \rightarrow {0},\,\, T \rightarrow {} {T_{\infty }}, \,\,{} C \rightarrow {} {C_{\infty }}, \,\,{} N \rightarrow {} {N_{\infty }} \,\, as \,\, z \rightarrow {\infty }, \end{aligned}$$

*u*(*r*, $$\vartheta $$, *z*), *v*(*r*, $$\vartheta $$, *z*) and *w*(*r*, $$\vartheta $$, *z*) have been designed for flow constitutients, *P* stands for pressure, magnetic field is presented by *B* = (0, 0, *B*$$ _{0} $$), the Hall parameter is *m*^42^. $$\lambda _{1}$$, $$\lambda _{2}$$ show the relaxation and retardation time parameters respectively. Similarly, $$\gamma _{1}$$ and $$\gamma _{2}$$ present thermal relaxation and mass relaxation time parameters respectively. Thermal diffusivity is $$\alpha $$, permeability of porous medium is *k*, Forchheimer resistance parameter is $$k^{F}$$ = $$\frac{C_{b}}{\sqrt{k}}$$, $$C_{b}$$ is the drag coefficient, Brownian diffusion coefficient is *D*$$_{B}$$, thermophoretic diffusion coefficient is *D*$$_{T}$$, $$ \rho _{f} $$ exhibits the density, $$ \mu _{f} $$ manifests the dynamic viscosity, $$ \sigma _{f} $$ enlightens the electrical conductivity and (*c*$$ _{p}) _{f} $$ represents heat capacity associated with Oldroyd-B nano-suspension. $$\nu _{f} = \frac{\mu _{f}}{\rho _{f}}$$ exhibits the kinematic viscousity, $$\tau = \frac{(\rho c)_{P}}{(\rho c)_{f}}$$ shows the quantity due to heat capacity, *b* employs the chemotaxis constant, *W*$$_{c}$$ exhibits the highest cell swimming speed and *D*$$_{n}$$ is the diffusivity of microorganisms.

The following transformations^18,27,46^ are introduced

9 $$\begin{aligned}{}&u = \textit{r}\Omega \textit{f}(\zeta ),\,\, \textit{v} = \textit{r}\Omega \textit{g}(\zeta ),\,\, \textit{w} = (\Omega \nu _{f})^{\frac{1}{2}}{} \textit{h}(\zeta ),\,\, \theta (\zeta ) = \frac{T-T_{\infty }}{T_{w}-T_{\infty }},\,\, \phi (\zeta ) = \frac{C-C_{\infty }}{C_{w}-C_{\infty }},\nonumber \\&\chi (\zeta ) = \frac{N-N_{\infty }}{N_{w}-N_{\infty }},\,\,\zeta = \begin{bmatrix}\frac{\Omega }{\nu _{f}}\end{bmatrix}^{\frac{1}{2}}z. \end{aligned}$$

Substituting the values from Eq. (9) in Eqs. (1–8), the below eight equations (10–17) are obtained.

10 $$\begin{aligned}{}&2f + h^{\prime } = 0, \end{aligned}$$

11 $$\begin{aligned}{}&f^{2} - g^{2} + f^{\prime }h - f^{\prime \prime } + \beta _{1}\begin{pmatrix}{h^{2}}{} f ^{\prime \prime } + 2f {f^{\prime }}h -2g g ^{\prime }h\end{pmatrix} + \beta _{2}\begin{pmatrix}2{f^{\prime }}^{2} + 2f ^{\prime }{} h ^{\prime \prime } - f ^{\prime \prime \prime }{} \textit{h}\end{pmatrix} \nonumber \\&\qquad - \dfrac{M(f^{\prime } - mg - 2\beta _{1}{} \textit{h}{f^{\prime }})}{1 + m^{2}} \nonumber \\&\qquad -\lambda _{3}f - \lambda _{4}f^{2} = 0, \end{aligned}$$

12 $$\begin{aligned}{}&2fg + {g^{\prime }}h - {g^{\prime \prime }} + \beta _{1}\begin{pmatrix}h^{2}{} g ^{\prime \prime } +2\begin{pmatrix}f g ^{\prime } + g {} f ^{\prime }\end{pmatrix}h\end{pmatrix} - \beta _{2}\begin{pmatrix}{} h {} g ^{\prime \prime \prime } - 2{f^{\prime }}{} g ^{\prime } - 2{h^{\prime \prime }}{g^{\prime }}\end{pmatrix} \nonumber \\&\qquad - \dfrac{M(mf^{\prime } + g - 2\beta _{1}{} \textit{h}{g^{\prime }})}{1 + m^{2}} \nonumber \\&\qquad -\lambda _{3}g - \lambda _{4}g^{2} = 0, \end{aligned}$$

13 $$\begin{aligned}{}&\frac{1}{Pr}\theta ^{\prime \prime } - h\theta ^{\prime } + Nb\begin{pmatrix}\theta ^{\prime }\phi ^{\prime } + \gamma _{3}\begin{pmatrix}h\theta ^{\prime }\phi ^{\prime \prime } + h\theta ^{\prime \prime }\phi ^{\prime }\end{pmatrix}\end{pmatrix} + Nt\begin{pmatrix}(\theta ^{\prime })^{2} + 2\gamma _{3}h\theta ^{\prime }\theta ^{\prime \prime }\end{pmatrix} \nonumber \\&\qquad - \gamma _{3}\begin{pmatrix}{h^{2}}\theta ^{\prime \prime } + h {h^{\prime }}\theta ^{\prime }\end{pmatrix} = 0, \end{aligned}$$

14 $$\begin{aligned}{}&\phi ^{\prime \prime } - Sc {} h \phi ^{\prime } - \gamma _{4}\begin{pmatrix}{h^{2}}\theta ^{\prime \prime } + h {h^{\prime }}\phi ^{\prime }\end{pmatrix} + \frac{Nt}{Nb}\begin{pmatrix}\theta ^{\prime \prime } + \gamma _{4}h\theta ^{\prime \prime \prime }\end{pmatrix} = 0, \end{aligned}$$

15 $$\begin{aligned}{}&\chi ^{\prime \prime } - Lbh\chi ^{\prime } - Pe \begin{pmatrix}\chi ^{\prime }\phi ^{\prime } + \phi ^{\prime \prime }(\gamma _{5} + \chi )\end{pmatrix} = 0, \end{aligned}$$

16 $$\begin{aligned}{}&f = \Omega _{1}, \,\, g = 1, \,\,{} h = 0, \,\, \theta = 1,\,\, \phi = 1,\,\,\chi = 1\,\, at \,\, \zeta = 0, \end{aligned}$$

17 $$\begin{aligned}{}&f \rightarrow {0}, \,\,{} g \rightarrow {0}, \,\,{} h \rightarrow {0}, \,\, \theta \rightarrow {0}, \,\,\phi \rightarrow {0}, \,\,\chi \rightarrow {0} \,\, as \,\, \zeta \rightarrow {\infty }, \end{aligned}$$

where (′) is applied for the derivative with respect to $\zeta$. $\beta _{1}$ = $\lambda _{1}$ $\Omega$ and $\beta _{2}$ = $\lambda _{2}$ $\Omega$ are the non-dimensional relaxation and retardation time parameters, $\gamma _{3}$ = $\gamma _{1}$ $\Omega$ is the non-dimensional thermal relaxation time parameter, $\gamma _{4}$ = $\gamma _{2}$ $\Omega$ is the non-dimensional solutal relaxation time parameter, $\Omega _{1}$ = $\frac{a}{\Omega }$ is the stretching parameter, $\lambda _{3}$ = $\frac{\nu _{f}}{k\Omega }$ is the porosity parameter, $\lambda _{4}$ = $\frac{k^{F}}{\sqrt{k}}$ is the Darcy Forchheimer parameter, *M* = $\frac{\sigma _{f} B^{2}_{0}}{\rho _{f}\Omega }$ is the magnetic field parameter, *Pr* = $\frac{\nu _{f}}{\alpha }$ denotes the Prandtl number, *Lb* = $\frac{\nu _{f}}{D_{n}}$ is the bioconvection Levis number, *Sc* = $\frac{\nu _{f}}{D_{B}}$ is the Schmidt number and *Pe* = $\frac{bW_{c}}{D_{n}}$ is the Peclet number, $\gamma _{5}$ = $\frac{N_{\infty }}{N_{w} - N_{\infty }}$ is the microorganisms concentration difference parameter, *Nb* = $\frac{\tau D_{B}(C_{w} - C_{\infty })}{\nu _{f}}$ is the Brownian motion parameter and *Nt* = $\frac{\tau D_{T}(T_{w} - T_{\infty })}{\nu _{f}T_{\infty }}$ represents the thermophoresis parameter.

$C_{F}$ (Skin friction coefficient), $Nu_{r}$ (local Nusselt), $Sh_{r}$ (Sherwood number) and $Nn_{r}$ (local motile density number), have the definitions

18 $$\begin{aligned} C_{F} = \frac{\tau |_{z=0}}{\rho _{f}(r\Omega )^{2}}, \end{aligned}$$

where

19 $$\begin{aligned} \tau = \sqrt{(\tau _{r})^{2} + (\tau _{\vartheta })^{2}}, \end{aligned}$$

exhibits the shear stress.

20 $$\begin{aligned} Nu_{r}=\frac{-rq_1}{\alpha (T_w-T_\infty )},\,\,Sh_{r}=\frac{-rq_2}{D_{B}(C_w-C_\infty )},\,\,Nn_{r}=\frac{-rq_3}{D_{m}(N_w-N_\infty )}, \end{aligned}$$

where $q_{1}$, $q_{2}$ and $q_{3}$ exhibit fluxes due to heat, mass and motile microorganisms having the formulations

21 $$\begin{aligned} q_{1}=-\alpha T_{z}|_{z=0},\,\,q_{2}=-D_{B}C_{z}|_{z=0},\,\,q_{3}=D_mN_z|_{z=0}. \end{aligned}$$

Upon employing the Eqs. (9), (18) goes to simplification as

22 $$\begin{aligned} C_{F} = Re_{r}^\frac{-1}{2}\begin{bmatrix}\begin{pmatrix}f^{\prime }(0)\end{pmatrix}^{2} + \begin{pmatrix}g^{\prime }(0)\end{pmatrix}^{2}\end{bmatrix}, \end{aligned}$$

where $Re_{r}$ = $\frac{r^{2}\Omega }{\nu _{f}}$ exhibits the Reynolds number.

Upon employing the Eq. (9) in Eq. (20), calculations are

23 $$\begin{aligned} Nu_{r}=-Re_{r}^{0.5}\theta ^{\prime }(0),\,\, Sh_{r}=-Re_{r}^{0.5}\phi ^{\prime }(0),\,\, Nn_{r}=-Re_{r}^{0.5}\chi ^{\prime }(0). \end{aligned}$$

## Computational work

Under the scenerio of HAM^52^, the initial approximations and auxiliary linear operators are

24 $$\begin{aligned}{}&h_{0}(\zeta ) = 0,{f_{0}}(\zeta ) = \Omega \exp (- \zeta ), g_{0}(\zeta ) = \exp (- \zeta ), \theta _{0}(\zeta ) = \exp (- \zeta ),\phi _{0}(\zeta ) = \exp (- \zeta ),\nonumber \\&\chi _{0}(\zeta ) = \exp (- \zeta ), \end{aligned}$$

25 $$\begin{aligned}{}&{{\varvec{L}}}_{h } = {h^{\prime }},  {{\varvec{L}}}_{f } = {f^{\prime \prime }} - f , {{\varvec{L}}}_{g } = {g^{\prime \prime }} - {g^\prime },  {{\varvec{L}}}_{\theta } = \theta ^{\prime \prime } - \theta , {{\varvec{L}}}_{\phi } = \phi ^{\prime \prime } - \phi ,  {{\varvec{L}}}_{\chi } = \chi ^{\prime \prime } - \chi . \end{aligned}$$

The linear operators are associated with

26 $$\begin{aligned} {{\varvec{L}}}_{h}\begin{bmatrix}{E_{1}}\end{bmatrix} = 0, \,\, {{\varvec{L}}}_{f}\begin{bmatrix}{E_{2}}\exp (\zeta ) + {E_{3}}\exp (- \zeta )\end{bmatrix} = 0, \,\, {{\varvec{L}}}_{g}\begin{bmatrix}{E_{4}}\exp (\zeta ) + {E_{5}}\exp (-\zeta )\end{bmatrix} = 0,\nonumber \\ {{\varvec{L}}}_{\theta }\begin{bmatrix}{E_{6}}\exp (\zeta ) + {E_{7}}\exp (-\zeta )\end{bmatrix} = 0, {{\varvec{L}}}_{\phi }\begin{bmatrix}{E_{8}}\exp (\zeta ) + {E_{9}}\exp (- \zeta )\end{bmatrix} = 0,\,\, {{\varvec{L}}}_{\chi }\begin{bmatrix}{E_{10}}\exp (\zeta ) + {E_{11}}\exp (- \zeta )\end{bmatrix} = 0, \end{aligned}$$

where *E*$_{i}$(*i* = 1-11) present the constants.

### Equations of deformation for zeroth order

Deformation equations of zeroth order are

27 $$\begin{aligned} (1 - q ) {\varvec{L}}_{h} [h (\zeta , q ) - {h_{0}}(\zeta )]= & {} q \hslash _{h} \aleph _{h} [f (\zeta , q ),h (\zeta , q)], \end{aligned}$$

28 $$\begin{aligned} (1 - q ) {\varvec{L}}_{f} [f (\zeta , q ) - {f_{0}}(\zeta )]= & {} q \hslash _{f} \aleph _{f} [f (\zeta , q ), g (\zeta , q ), h (\zeta , q)], \end{aligned}$$

29 $$\begin{aligned} (1 - q ) {\varvec{L}}_{g} [g (\zeta , q ) - {g_{0}}(\zeta )]= & {} q \hslash _{g} \aleph _{g} [f (\zeta , q ), g (\zeta , q ), h (\zeta , q)], \end{aligned}$$

30 $$\begin{aligned} (1 - q ) {\varvec{L}}_{\theta } [\theta (\zeta , q ) - \theta _{0}(\zeta )]= & {} q \hslash _{\theta } \aleph _{\theta } [h (\zeta , q), \theta (\zeta , q ), \phi (\zeta , q )], \end{aligned}$$

31 $$\begin{aligned} (1 - q ) {\varvec{L}}_{\phi } [\phi (\zeta , q ) - \phi _{0}(\zeta )]= & {} q \hslash _{\phi } \aleph _{\phi } [h (\zeta , q), \theta (\zeta , q ), \phi (\zeta , q )], \end{aligned}$$

32 $$\begin{aligned} (1 - q ) {\varvec{L}}_{\chi } [\chi (\zeta , q ) - \chi _{0}(\zeta )]= & {} q \hslash _{\chi } \aleph _{\chi } [h (\zeta , q), \chi (\zeta , q ), \phi (\zeta , q )], \end{aligned}$$

where $$q $$ is presented as an embedding parameter and $$\hslash _{f}$$, $$\hslash _{g}$$, $$\hslash _{h}$$, $$\hslash _{\theta }$$, $$\hslash _{\phi }$$ and $$\hslash _{\chi }$$ are the non-zero auxiliary parameters. The nonlinear operators $$\aleph _{f}$$, $$\aleph _{g}$$, $$\aleph _{h}$$, $$\aleph _{\theta }$$, $$\aleph _{\phi }$$, and $$\aleph _{\chi }$$ are defined as

33 $$\begin{aligned} \aleph _{h}[f(\zeta , q ), h (\zeta , q)]= & {} 2{f(\zeta , {q})} + \frac{\partial h(\zeta , q)}{\partial \zeta }, \end{aligned}$$

34 $$\begin{aligned} \aleph _{f}[f(\zeta , q ), g(\zeta , q ), h(\zeta , q ),\theta (\zeta , q )]= & {} - \frac{\partial ^{2}f(\zeta , q )}{\partial \zeta ^{2}} + \begin{pmatrix}f(\zeta , q )\end{pmatrix}^{2} - \begin{pmatrix}g(\zeta , q )\end{pmatrix}^{2} - \frac{\partial f(\zeta , q)}{\partial \zeta }{h(\zeta , {q})} \nonumber \\&+\beta _{1}\begin{bmatrix}\begin{pmatrix}h(\zeta , q )\end{pmatrix}^{2}\frac{\partial ^{2}f(\zeta , q )}{\partial \zeta ^{2}} + 2f(\zeta , q )\frac{\partial f(\zeta , q )}{\partial \zeta }h(\zeta , q ) - 2g(\zeta , q )\frac{\partial g(\zeta , q )}{\partial \zeta }h(\zeta , q )\end{bmatrix} \nonumber \\&+\beta _{2}\begin{bmatrix}2\begin{pmatrix}\frac{\partial f(\zeta , q )}{\partial \zeta }\end{pmatrix}^{2} + 2\frac{\partial f(\zeta , q )}{\partial \zeta }\frac{\partial ^{2} h(\zeta , q )}{\partial \zeta ^{2}} - \frac{\partial ^{3}f(\zeta , q )}{\partial \zeta ^{3}}h(\zeta , q )\end{bmatrix} \nonumber \\&- \dfrac{M}{1 + m^{2}}\begin{bmatrix}\frac{\partial f(\zeta , q )}{\partial \zeta } - mg(\zeta , q ) -2\beta _{1}h(\zeta , q )\frac{\partial f(\zeta , q)}{\partial \zeta }\end{bmatrix}\nonumber \\&- \lambda _{3}f(\zeta , q ) - \lambda _{4}\begin{pmatrix}f(\zeta , q )\end{pmatrix}^{2}, \end{aligned}$$

35 $$\begin{aligned} \aleph _{g}[f(\zeta , q ), g(\zeta , q ), h(\zeta , q )]= & {} 2f(\zeta , q )g(\zeta , q ) + \frac{\partial g(\zeta , q)}{\partial \zeta }{h(\zeta , {q})} - \frac{\partial ^{2}g(\zeta , q )}{\partial \zeta ^{2}} \nonumber \\&+\beta _{1}\begin{bmatrix}\begin{pmatrix}h(\zeta , q )\end{pmatrix}^{2}\frac{\partial ^{2}g(\zeta , q )}{\partial \zeta ^{2}} + 2f(\zeta , q )\frac{\partial g(\zeta , q )}{\partial \zeta }h(\zeta , q ) + 2g(\zeta , q )\frac{\partial f(\zeta , q )}{\partial \zeta }h(\zeta , q )\end{bmatrix} \nonumber \\&-\beta _{2}\begin{bmatrix}h(\zeta , q )\frac{\partial ^{3}g(\zeta , q )}{\partial \zeta ^{3}} - 2\frac{\partial f(\zeta , q )}{\partial \zeta }\frac{\partial g(\zeta , q )}{\partial \zeta } - 2\frac{\partial ^{2}h(\zeta , q )}{\partial \zeta ^{2}}\frac{\partial g(\zeta , q )}{\partial \zeta }\end{bmatrix} \nonumber \\&- \dfrac{M}{1 + m^{2}}\begin{bmatrix}\frac{m \partial f(\zeta , q )}{\partial \zeta } + g(\zeta , q ) - 2\beta _{1}h(\zeta , q )\frac{\partial g(\zeta , q)}{\partial \zeta }\end{bmatrix}\nonumber \\&- \lambda _{3}g(\zeta , q ) - \lambda _{4}\begin{pmatrix}g(\zeta , q )\end{pmatrix}^{2}, \end{aligned}$$

36 $$\begin{aligned} \aleph _{\theta }[f(\zeta , q), h(\zeta , q), \theta (\zeta , q), \phi (\zeta , q)]= & {} \frac{1}{Pr}\frac{\partial ^{2}\theta (\zeta , q)}{\partial \zeta ^{2}} - h(\zeta , q )\frac{\partial \theta (\zeta , q)}{\partial \zeta } \nonumber \\&+Nb\begin{bmatrix}\frac{\partial \theta (\zeta , q)}{\partial \zeta }\frac{\partial \phi (\zeta , q)}{\partial \zeta } + \gamma _{3}\begin{pmatrix} h(\zeta , q)\frac{\partial \theta (\zeta , q)}{\partial \zeta }\frac{\partial ^{2} \phi (\zeta , q)}{\partial \zeta ^{2}}+ h(\zeta , q)\frac{\partial ^{2}\theta (\zeta , q)}{\partial \zeta ^{2}}\frac{\partial \phi (\zeta , q)}{\partial \zeta }\end{pmatrix} \end{bmatrix} \nonumber \\&+ Nt\begin{bmatrix}\begin{pmatrix}\frac{\partial \theta (\zeta , q)}{\partial \zeta }\end{pmatrix}^{2} + 2\gamma _{3} h(\zeta , q)\frac{\partial \theta (\zeta , q)}{\partial \zeta }\frac{\partial ^{2}\theta (\zeta , q)}{\partial \zeta ^{2}}\end{bmatrix}\nonumber \\&- \gamma _{3}\begin{bmatrix}\begin{pmatrix}h(\zeta , q )\end{pmatrix}^{2}\frac{\partial ^{2}\theta (\zeta , q)}{\partial \zeta ^{2}} + h(\zeta , q )\frac{\partial h(\zeta , q )}{\partial \zeta }\frac{\partial \theta (\zeta , q)}{\partial \zeta }\end{bmatrix}, \end{aligned}$$

37 $$\begin{aligned} \aleph _{\phi }[h(\zeta , q), \theta (\zeta , q), \phi (\zeta , q)]= & {} \frac{\partial ^{2} \phi (\zeta , q)}{\partial \zeta ^{2}} - \textit{Sc} h(\zeta , q)\frac{\partial \phi (\zeta , q)}{\partial \zeta } \nonumber \\&- \gamma _{4}\begin{bmatrix}\begin{pmatrix}h(\zeta , q )\end{pmatrix}^{2}\frac{\partial ^{2}\theta (\zeta , q)}{\partial \zeta ^{2}} + h(\zeta , q )\frac{\partial h(\zeta , q )}{\partial \zeta }\frac{\partial \phi (\zeta , q)}{\partial \zeta }\end{bmatrix}\nonumber \\&+ \frac{Nt}{Nb}\begin{pmatrix}\frac{\partial ^{2}\theta (\zeta , q)}{\partial \zeta ^{2}} + \gamma _{4}h(\zeta , q )\frac{\partial ^{3}\theta (\zeta , q)}{\partial \zeta ^{3}}\end{pmatrix}, \end{aligned}$$

38 $$\begin{aligned} \aleph _{\chi }[h(\zeta , q), \phi (\zeta , q), \chi (\zeta , q)]= & {} \frac{\partial ^{2} \chi (\zeta , q)}{\partial \zeta ^{2}} - Lb h(\zeta , q)\frac{\partial \chi (\zeta , q)}{\partial \zeta } \nonumber \\&- Pe \begin{bmatrix}\frac{\partial \phi (\zeta , q)}{\partial \zeta }\frac{\partial \chi (\zeta , q)}{\partial \zeta } + \frac{\partial ^{2} \phi (\zeta , q)}{\partial \zeta ^{2}}\begin{pmatrix}\gamma _{5} + \chi (\zeta , q)\end{pmatrix}\end{bmatrix}, \end{aligned}$$

Eq. (27) retains the B.C.

39 $$\begin{aligned} h (0, q) = 0. \end{aligned}$$

Eq. (28) retains B.C.

40 $$\begin{aligned} f (0, q) = \Omega _{1},\,\, f (\infty , q) = 0. \end{aligned}$$

Eq. (29) retains B.C.

41 $$\begin{aligned} g (0, q) = 1,\,\, g (\infty , q) = 0. \end{aligned}$$

Eq. (30) retains B.C.

42 $$\begin{aligned} \theta (0, q) = 1,\,\, {\theta } (\infty , q) = 0. \end{aligned}$$

Eq. (31) retains B.C.

43 $$\begin{aligned} \phi (0, q) = 1, \phi (\infty , q) = 0. \end{aligned}$$

Eq. (32) retains B.C.

44 $$\begin{aligned} \chi (0, q) = 1, \chi (\infty , q) = 0. \end{aligned}$$

Upon *q* = 0 and *q* = 1, Eqs. (27–32) become

45 $$\begin{aligned}{}&q = 0 \Rightarrow h (\zeta , 0) = h_{0}(\zeta )  and  q = 1 \Rightarrow h (\zeta , 1) = h(\zeta ), \end{aligned}$$

46 $$\begin{aligned}{}&q = 0 \Rightarrow f (\zeta , 0) = f_{0}(\zeta )  and  q = 1 \Rightarrow f (\zeta , 1) = f(\zeta ), \end{aligned}$$

47 $$\begin{aligned}{}&q = 0 \Rightarrow g (\zeta , 0) = g_{0}(\zeta )  and  q = 1 \Rightarrow g (\zeta , 1) = g(\zeta ), \end{aligned}$$

48 $$\begin{aligned}{}&q = 0 \Rightarrow \theta (\zeta , 0) = \theta _{0}(\zeta )  and  q = 1 \Rightarrow \theta (\zeta , 1) = \theta (\zeta ), \end{aligned}$$

49 $$\begin{aligned}{}&q = 0 \Rightarrow \phi (\zeta , 0) = \phi _{0}(\zeta )  and  q = 1 \Rightarrow \phi (\zeta , 1) = \phi (\zeta ), \end{aligned}$$

50 $$\begin{aligned}{}&q = 0 \Rightarrow \chi (\zeta , 0) = \chi _{0}(\zeta )  and  q = 1 \Rightarrow \chi (\zeta , 1) = \chi (\zeta ). \end{aligned}$$

Expanding *h*($\zeta$, *q*), *f*($\zeta$, *q*), *g*($\zeta$, *q*), $\theta$($\zeta$, *q*), $\phi$($\zeta$, *q*) and $\chi$($\zeta$, *q*) through Taylor series, Eqs. (45–50) generate

51 $$\begin{aligned}{}&h (\zeta , q ) = {h_{0}}(\zeta ) + \sum ^{\infty }_{m = 1} {h_{m}}(\zeta ){q^{m}}, \quad {h_{m}}(\zeta ) = \frac{1}{m!} \frac{\partial ^{m} h (\zeta , q )}{\partial \zeta ^{m}}\mid _{q=0}, \end{aligned}$$

52 $$\begin{aligned}{}&f (\zeta , q ) = {f_{0}}(\zeta ) + \sum ^{\infty }_{m = 1} {f_{m}}(\zeta ){q^{m}}, \quad {f_{m}}(\zeta ) = \frac{1}{m!} \frac{\partial ^{m} f (\zeta , q )}{\partial \zeta ^{m}}\mid _{q=0}, \end{aligned}$$

53 $$\begin{aligned}{}&g (\zeta , q ) = {g_{0}}(\zeta ) + \sum ^{\infty }_{m = 1} {g_{m}}(\zeta ){q^{m}}, \quad {g_{m}}(\zeta ) = \frac{1}{m!} \frac{\partial ^{m} g (\zeta , q )}{\partial \zeta ^{m}}\mid _{q=0}, \end{aligned}$$

54 $$\begin{aligned}{}&\theta (\zeta , q ) = {\theta _{0}}(\zeta ) + \sum ^{\infty }_{m = 1} {\theta _{m}}(\zeta ){q^{m}}, \quad {\theta _{m}}(\zeta ) = \frac{1}{m!} \frac{\partial ^{m} \theta (\zeta , q )}{\partial \zeta ^{m}}\mid _{q=0}, \end{aligned}$$

55 $$\begin{aligned}{}&\phi (\zeta , q ) = \phi _{0}(\zeta ) + \sum ^{\infty }_{m = 1} \phi (\zeta ){q^{m}}, \quad \phi (\zeta ) = \frac{1}{m!} \frac{\partial ^{m} \phi (\zeta , q )}{\partial \zeta ^{m}}\mid _{q=0}, \end{aligned}$$

56 $$\begin{aligned}{}&\chi (\zeta , q ) = {\chi _{0}}(\zeta ) + \sum ^{\infty }_{m = 1} {\chi _{m}}(\zeta ){q^{m}}, \quad {\chi _{m}}(\zeta ) = \frac{1}{m!} \frac{\partial ^{m} \chi (\zeta , q )}{\partial \zeta ^{m}}\mid _{q=0}. \end{aligned}$$

From Eqs. (51–56), the convergence of the series is obtained by taking *q* = 1 for the appropriate values of $\hslash _{f}$, $\hslash _{g}$, $\hslash _{h}$, $\hslash _{\theta }$, $\hslash _{\phi }$ and $\hslash _{\chi }$, so

57 $$\begin{aligned} h (\zeta )= & {} {h_{0}}(\zeta ) + \sum ^{\infty }_{m = 1}{h_{m}}(\zeta ), \end{aligned}$$

58 $$\begin{aligned} f (\zeta )= & {} {f_{0}}(\zeta ) + \sum ^{\infty }_{m = 1}{f_{m}}(\zeta ), \end{aligned}$$

59 $$\begin{aligned} g (\zeta )= & {} {g_{0}}(\zeta ) + \sum ^{\infty }_{m = 1}{g_{m}}(\zeta ), \end{aligned}$$

60 $$\begin{aligned} \theta (\zeta )= & {} \theta _{0}(\zeta ) + \sum ^{\infty }_{m = 1}{\theta _{m}}(\zeta ), \end{aligned}$$

61 $$\begin{aligned} \phi (\zeta )= & {} \phi _{0}(\zeta ) + \sum ^{\infty }_{m = 1}\phi (\zeta ), \end{aligned}$$

62 $$\begin{aligned} \chi (\zeta )= & {} \chi _{0}(\zeta ) + \sum ^{\infty }_{m = 1}\chi (\zeta ). \end{aligned}$$

### Deformation equations of *m*-th order

Equations having *m*-th order deformations become

63 $$\begin{aligned} {\varvec{L}}_{h}[{h_{m}}(\zeta ) - \psi _{m}{h_{m-1}}(\zeta )]= & {} \hslash _{h}\mathfrak {R}_{m}^{h}(\zeta ), \end{aligned}$$

64 $$\begin{aligned} {\varvec{L}}_{f}[{f_{m}}(\zeta ) - \psi _{m}{f_{m-1}}(\zeta )]= & {} \hslash _{f}\mathfrak {R}_{m}^{f}(\zeta ), \end{aligned}$$

65 $$\begin{aligned} {\varvec{L}}_{g}[{g_{m}}(\zeta ) - \psi _{m}{g_{m-1}}(\zeta )]= & {} \hslash _{g}\mathfrak {R}_{m}^{g}(\zeta ), \end{aligned}$$

66 $$\begin{aligned} {{\varvec{L}}}_\theta [\theta _{m}(\zeta ) - \psi _{m}\theta _{m-1}(\zeta )]= & {} \hslash _{\theta }\mathfrak {R}_{m}^{\theta }(\zeta ), \end{aligned}$$

67 $$\begin{aligned} {{\varvec{L}}}_\phi [\phi _{m}(\zeta ) - \psi _{m}\phi _{m-1}(\zeta )]= & {} \hslash _{\phi }\mathfrak {R}_{m}^{\phi }(\zeta ), \end{aligned}$$

68 $$\begin{aligned} {{\varvec{L}}}_\chi [\chi _{m}(\zeta ) - \psi _{m}\chi _{m-1}(\zeta )]= & {} \hslash _{\chi }\mathfrak {R}_{m}^{\chi }(\zeta ), \end{aligned}$$

69 $$\begin{aligned}{}&h_{m}(0) = 0, \end{aligned}$$

70 $$\begin{aligned}{}&f_{m}(0) = 0, f_{m}(\infty ) = 0, \end{aligned}$$

71 $$\begin{aligned}{}&g_{m}(0) = 0, g_{m}(\infty ) = 0, \end{aligned}$$

72 $$\begin{aligned}{}&\theta _{m}(0) = 0, \theta _{m}(\infty ) = 0, \end{aligned}$$

73 $$\begin{aligned}{}&\phi _{m}(0) = 0, \phi _{m}(\infty ) = 0, \end{aligned}$$

74 $$\begin{aligned}{}&\chi _{m}(0) = 0, \chi _{m}(\infty ) = 0, \end{aligned}$$

where

75 $$\begin{aligned} \mathfrak {R}_{m}^{h}(\zeta )= & {} h^{\prime }_{m-1} + 2f_{m-1}, \end{aligned}$$

76 $$\begin{aligned} \mathfrak {R}_{m}^{f}(\zeta )= & {} - f^{\prime \prime }_{m-1} + \sum ^{m - 1}_{k = o}f_{m - 1 - k} f_{k} - \sum ^{m - 1}_{k = o}g_{m - 1 - k}g_{k} + \sum ^{m - 1}_{k = o}f^{\prime }_{m - 1 - k}h_{k} \nonumber \\&+\beta _{1}\begin{bmatrix}\sum ^{m - 1}_{k = o}h_{m-1-k} \sum ^{k}_{l = o}h_{k-l}f^{\prime \prime }_{l} + 2\sum ^{m - 1}_{k = o}f_{m - 1 - k} \sum ^{k}_{l = o}f^{\prime }_{k-l}h_{l} - 2\sum ^{m - 1}_{k = o}g_{m - 1 - k} \sum ^{k}_{l = o}g^{\prime }_{k-l}h_{l}\end{bmatrix}\nonumber \\&+ \beta _{2}\sum ^{m - 1}_{k = o}\begin{bmatrix}2f^{\prime }_{m - 1 - k} f_{k} + 2f^{\prime }_{m - 1 - k} h^{\prime \prime }_{k} - f^{\prime \prime \prime }_{m - 1 - k} h_{k}\end{bmatrix} \nonumber \\&- \dfrac{M}{1 + m^{2}}\begin{bmatrix} f^{\prime }_{m - 1} - m {} g _{m-1} - 2\beta _{1} \sum ^{m - 1}_{k = o}h_{m - 1 - k}f^{\prime }_{k}\end{bmatrix}\nonumber \\&\lambda _{3}f_{m - 1} - \lambda _{4} \sum ^{m - 1}_{k = o}f_{m - 1 - k} f_{k}, \end{aligned}$$

77 $$\begin{aligned} \mathfrak {R}_{m}^{g}(\zeta )= & {} - g^{\prime \prime }_{m-1} + \sum ^{m - 1}_{k = o} g^{\prime }_{m - 1 - k} h_{k} + 2\sum ^{m - 1}_{k = o}f_{m - 1 - k} g_{k} \nonumber \\&+\beta _{1}\begin{bmatrix}\sum ^{m - 1}_{k = o}h_{m-1-k} \sum ^{k}_{l = o}h_{k-l}g^{\prime \prime }_{l} + 2\sum ^{m - 1}_{k = o}f_{m - 1 - k} \sum ^{k}_{l = o}g^{\prime }_{k-l}h_{l} + 2\sum ^{m - 1}_{k = o}g_{m - 1 - k} \sum ^{k}_{l = o}f^{\prime }_{k-l}h_{l}\end{bmatrix}\nonumber \\&- \beta _{2}\sum ^{m - 1}_{k = o}\begin{bmatrix}h_{m - 1 - k} g^{\prime \prime \prime }_{k} - 2f^{\prime }_{m - 1 - k} g^{\prime }_{k} - 2h^{\prime \prime }_{m - 1 - k} g^{\prime }_{k}\end{bmatrix} \nonumber \\&- \dfrac{M}{1 + m^{2}}\begin{bmatrix} m f^{\prime }_{m - 1} + g _{m-1} - 2\beta _{1} \sum ^{m - 1}_{k = o}h_{m - 1 - k}g^{\prime }_{k} \end{bmatrix}\nonumber \\&\lambda _{3}g_{m - 1} - \lambda _{4} \sum ^{m - 1}_{k = o}g_{m - 1 - k} g_{k}, \end{aligned}$$

78 $$\begin{aligned} \mathfrak {R}_{m}^{\theta }(\zeta )= & {} \frac{1}{Pr}\theta ^{\prime \prime }_{m-1} - \sum ^{m - 1}_{k = o} h_{m - 1 - k} \theta ^{\prime }_{k} \nonumber \\&+Nb\begin{bmatrix}\sum ^{m - 1}_{k = o}\theta ^{\prime }_{m-1-k}\phi ^{\prime }_{k} + \gamma _{3}\begin{pmatrix}\sum ^{m - 1}_{k = o}h_{m - 1 - k} \sum ^{k}_{l = o}\theta ^{\prime }_{k-l}\phi ^{\prime \prime }_{l} + \sum ^{m - 1}_{k = o}h_{m - 1 - k} \sum ^{k}_{l = o}\theta ^{\prime \prime }_{k-l}\phi ^{\prime }_{l}\end{pmatrix}\end{bmatrix}\nonumber \\&+ Nt\begin{bmatrix}\sum ^{m - 1}_{k = o}\theta ^{\prime }_{m-1-k}\theta ^{\prime }_{k} + 2\gamma _{3}\sum ^{m - 1}_{k = o}h_{m - 1 - k} \sum ^{k}_{l = o}\theta ^{\prime }_{k-l}\theta ^{\prime \prime }_{l}\end{bmatrix} \nonumber \\&- \gamma _{3}\sum ^{m - 1}_{k = o}h_{m - 1 - k} \sum ^{k}_{l = o}\begin{bmatrix}h_{k-l}\theta ^{\prime \prime }_{l} + h^{\prime }_{k-l}\theta ^{\prime }_{l}\end{bmatrix}, \end{aligned}$$

79 $$\begin{aligned} \mathfrak {R}^{\phi }_{m}(\zeta )= & {} \phi ^{\prime \prime }_{m-1} - Sc\sum ^{m - 1}_{k = o}h_{m - 1 - k}\phi ^{\prime }_{k} - \gamma _{4}\sum ^{m - 1}_{k = o}h_{m - 1 - k} \sum ^{k}_{l = o}\begin{bmatrix}h_{k-l}\theta ^{\prime \prime }_{l} + h^{\prime }_{k-l}\phi ^{\prime }_{l}\end{bmatrix} \nonumber \\&+ \frac{Nt}{Nb}\begin{bmatrix}\theta ^{\prime \prime }_{m-1} + \gamma _{4}\sum ^{m - 1}_{k = o} h_{m - 1 - k} \theta ^{\prime \prime \prime }_{k}\end{bmatrix}, \end{aligned}$$

80 $$\begin{aligned} \mathfrak {R}^{\chi }_{m}(\zeta )= & {} \chi ^{\prime \prime }_{m-1} - Lb \sum ^{m - 1}_{k = o}h_{m - 1 - k}\chi ^{\prime }_{k} - Pe \sum ^{m - 1}_{k = o}\begin{bmatrix}\chi ^{\prime }_{m-1-k}\phi ^{\prime }_{k} + \phi ^{\prime \prime }_{m-1-k}\chi _{k}\end{bmatrix} - \gamma _{5}{} Pe \phi ^{\prime \prime }_{m}, \end{aligned}$$

81 $$\begin{aligned} \psi _{m}= & {} \left\{ \begin{array}{ll} 0, &{} {{m} \leqslant 1} \\ 1, &{} {{m} > 1.} \end{array} \right. \end{aligned}$$

For the particular solutions *h*$^{*}_{m}$($\zeta$), *f*$^{*}_{m}$($\zeta$), *g*$^{*}_{m}$($\zeta$), $\theta ^{*}_{m}$($\zeta$), $\phi ^{*}_{m}$($\zeta$) and $\chi ^{*}_{m}$($\zeta$), the general evaluations of Eqs. (63–68) are

82 $$\begin{aligned} {h_{m}}(\zeta )= & {} {h^{*}_{m}}(\zeta ) + {E_{1}}, \end{aligned}$$

83 $$\begin{aligned} {f_{m}}(\zeta )= & {} {f^{*}_{m}}(\zeta ) + {E_{2}}\exp (-\zeta ) + {E_{3}}\exp (\zeta ), \end{aligned}$$

84 $$\begin{aligned} {g_{m}}(\zeta )= & {} {g^{*}_{m}}(\zeta ) + {E_{4}}\exp (-\zeta ) + {E_{5}}\exp (\zeta ), \end{aligned}$$

85 $$\begin{aligned} \theta _{m}(\zeta )= & {} \theta ^{*}_{m}(\zeta ) + {E_{6}}\exp (-\zeta ) + {E_{7}}\exp (\zeta ), \end{aligned}$$

86 $$\begin{aligned} \phi _{m}(\zeta )= & {} \phi ^{*}_{m}(\zeta ) + {E_{8}}\exp (-\zeta ) + {E_{9}}\exp (\zeta ), \end{aligned}$$

87 $$\begin{aligned} \chi _{m}(\zeta )= & {} \chi ^{*}_{m}(\zeta ) + {E_{10}}\exp (-\zeta ) + {E_{11}}\exp (\zeta ). \end{aligned}$$

## Comparsion of authors computations to existing values

Data is given in Table 1 for the authentication of authors work.

**Table 1:** Data presented as the comparsion of author’s computations.

Profiles	Waqas et al.^41^	Author’s computations
*f*′(0)	0.5101162642	0.5101162641
*g*′(0)	0.6158492795	0.6158492796
θ′(0)	0.933641126	0.933641125

## Results and discussion

**Figure 2:**
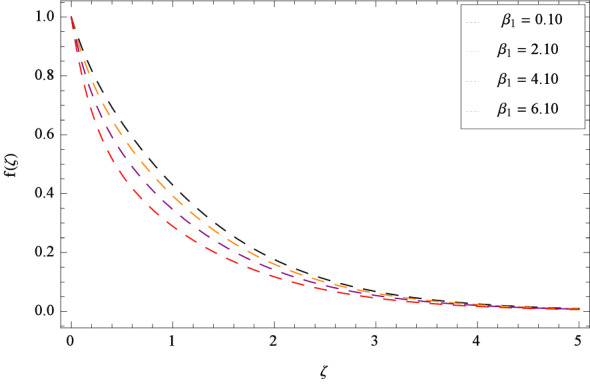
Pattern of graph curves on account of $\beta _{1}$.

**Figure 3:**
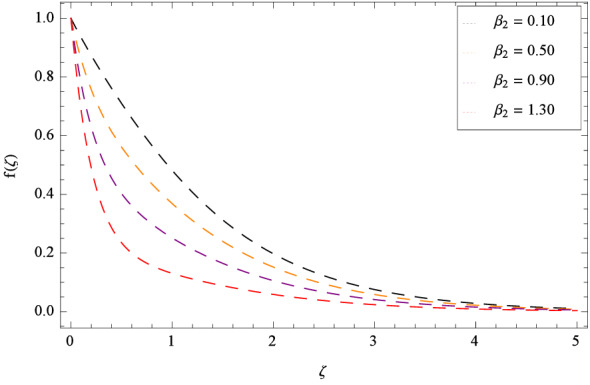
Pattern of graph curves on account of $\beta _{2}$.

**Figure 4:**
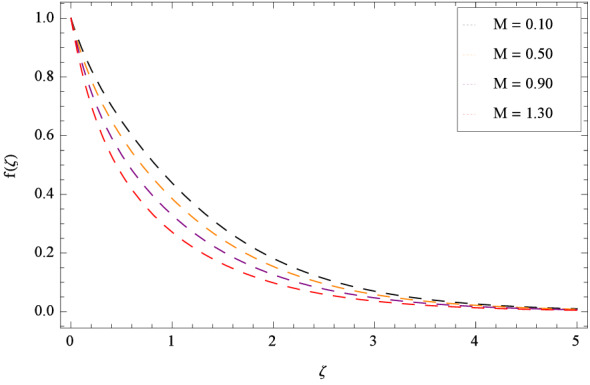
Pattern of graph curves on account of *M*.

**Figure 5:**
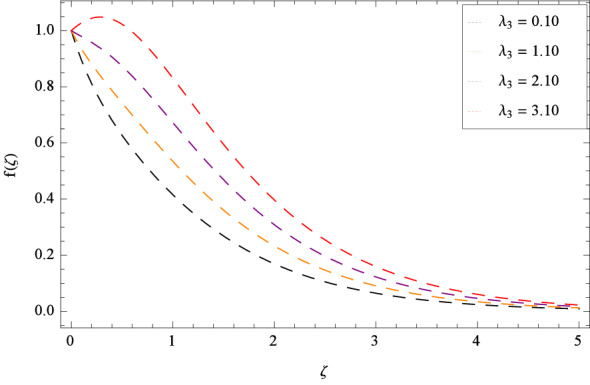
Pattern of graph curves on account of $\lambda _{3}$.

**Figure 6:**
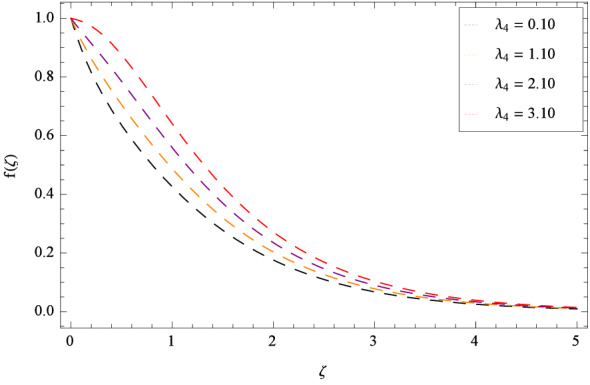
Pattern of graph curves on account of $\lambda _{4}$.

**Figure 7:**
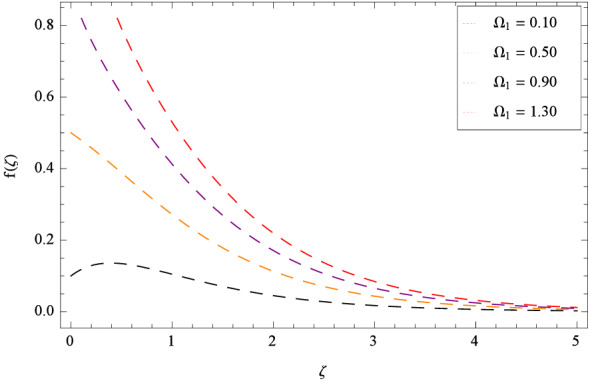
Pattern of graph curves on account of $\Omega _{1}$.

In Fig. 2, $\beta _{1}$ shows the effect of non-dimensional relaxation time parameter on radial velocity *f*($\zeta$) which tends to decrement. As the relaxation time parameter is the ratio of material relaxation time to the material observation time. So it is physically justified that for the greater values of $\beta _{1}$, the high values of relaxation time address that the liquid remains solid-like in major due to which the fluid flow decreases in each side. For the effect of retardation time parameter $\beta _{2}$ on radial velocity *f*($\zeta$), Fig. 3 is sketched which remarks that the magnitude of flow is decelerated. Retardation time shows the time spent on the generation of shear stress in a fluid. So it presents the time-scales noted during the start-up experiments that are not interpreted through relaxation time. $\beta _{1} = 0$ and $\beta _{2} = 0$ correspond to the study of viscous nanofluid. Figure 4 depicts that the radial velocity *f*($\zeta$) is decelerated due to magnetic field parameter *M*. The interface of strong magnetic field represents remarkable decay of the flow of fluid. As *M* is assuming the high values, the Lorentz forces come into play which reduce the liquid flow. The existence of magnetic field challenge the flowing status and finally decelerates the radial velocity. So magnetohydrodynamics is a procedure which control the fluid motion. *M* = 0, shows the absence of magnetohydrodynamic effect. Porosity parameter $\lambda _{3}$ enhances the flow function of fluid as shown in Fig. 5. Similarly the Darcy Forchheimer parameter $\lambda _{4}$ highlightes the flow of the Oldroyd-B nanofluid which is shown in Fig. 6. The stretching parameter $\Omega _{1}$ effect is shown in Fig. 7. It is noted that for the greater values of the stretching parameter $\Omega _{1}$, the radial velocity *f*($\zeta$) is enhanced since the stretching phenomena increases in radial direction i.e. uplifting behavior is seen for the radial velocity for the stretching values of $\Omega _{1}$.

**Figure 8:**
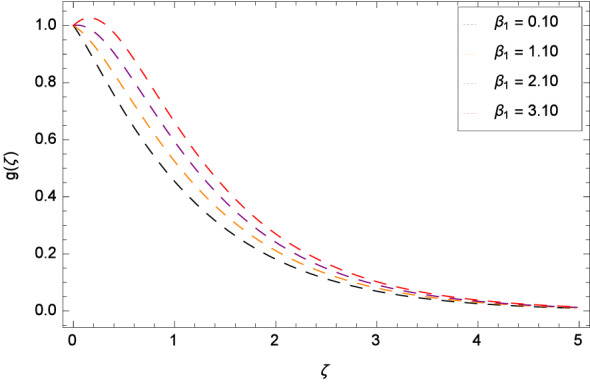
Pattern of graph curves on account of $\beta _{1}$.

**Figure 9:**
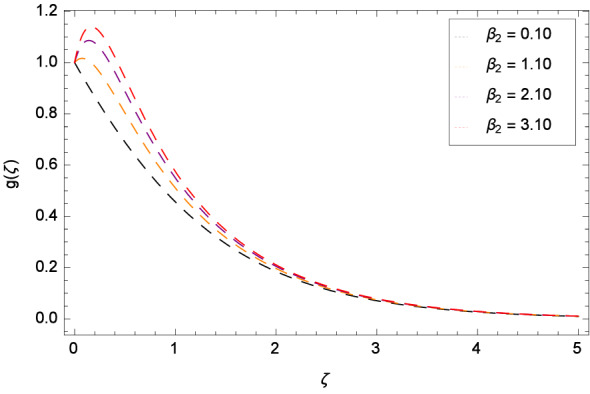
Pattern of graph curves on account of $\beta _{2}$.

**Figure 10:**
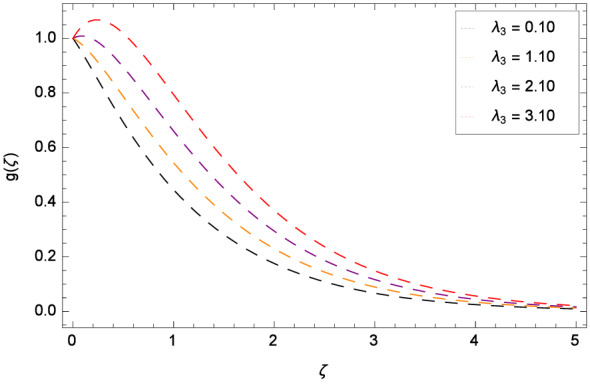
Pattern of graph curves on account of $\lambda _{3}$.

**Figure 11:**
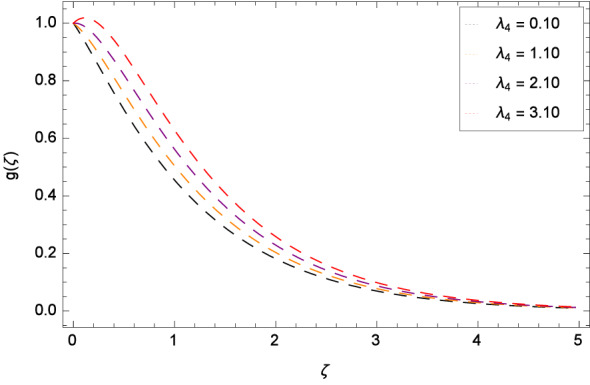
Pattern of graph curves on account of $\lambda _{4}$.

**Figure 12:**
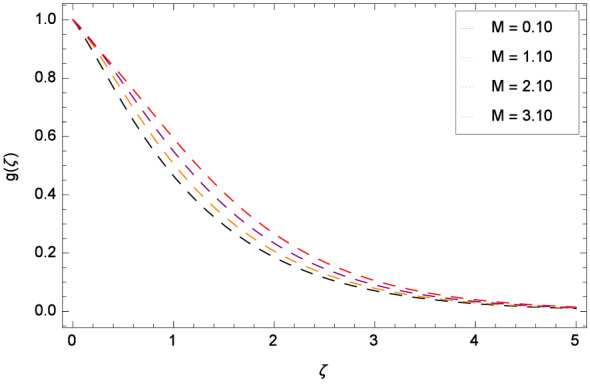
Pattern of graph curves on account of *M*.

Figure 8 reports that azimuthal velocity *g*($\zeta$) has high magnitude for the non-dimensional relaxation time parameter $\beta _{1}$. It is noted that the flow is enhanced in the negative direction. Physically, during rotation, the disk is playing the role of centrifugal pump which pushes the fluid in the outward direction. Figure 9 displays the impact of the non-dimensional retardation time parameter $\beta _{2}$ on the azimuthal velocity *g*($\zeta$) which enhances for the variation of $\beta _{2}$ through the values 0.10, 1.10, 2.10 & 3.10. Physically, retardation time shows the time spent on designing the shear stress in the Oldroyd-B nanofluid. There exists a direct relation of retardation time parameter $\beta _{2}$ and azimuthal velocity *g*($\zeta$). It is concluded that fluid flowing along the disk is maximum. Figures 10 and 11 remark that azimuthal velocity *g*($\zeta$) is on the high position for the various values of porosity parameter $\lambda _{3}$ and Darcy Forchheimer parameter $\lambda _{4}$ respectively. Figure 12 is drawn to understand the influence magnetohydrodynamics on azimuthal velocity *g*($\zeta$). The azimuthal velocity *g*($\zeta$) is made strong in flow due to the high external applied magnetic field.

**Figure 13:**
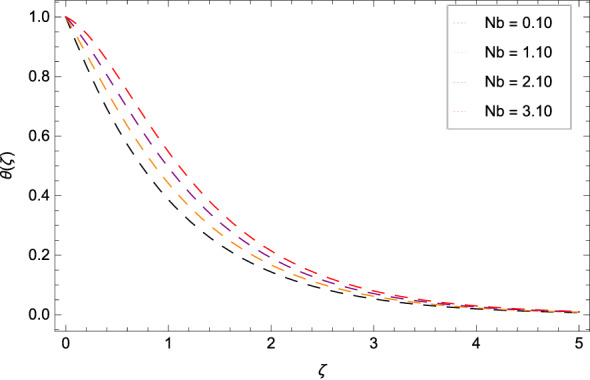
Pattern of graph curves on account of *Nb*.

**Figure 14:**
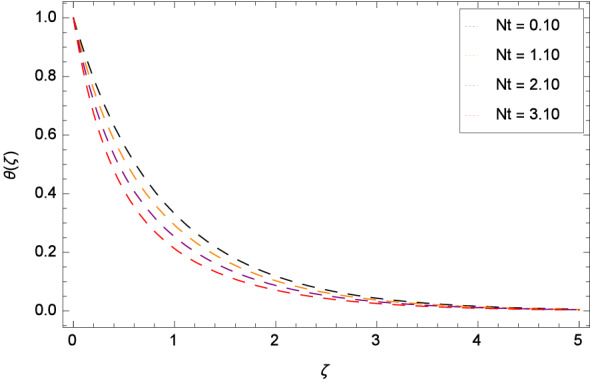
Pattern of graph curves on account of *Nt*.

**Figure 15:**
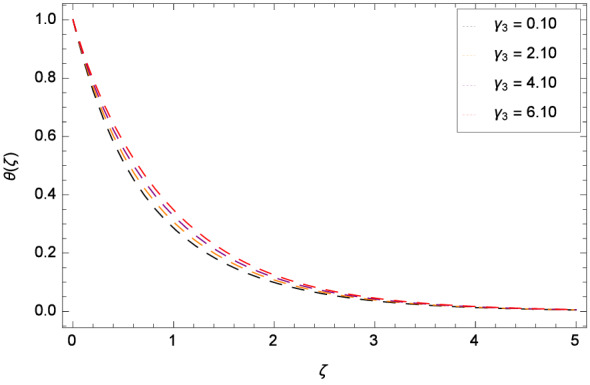
Pattern of graph curves on account of $\gamma _{3}$.

Figure 13 depicts that the temperature is increased for the high values of Brownian motion parameter *Nb*. The fact is that a higher rate of *Nb* yields the higher diffusion. Due to Brownian motion, the extra energy is generated which in turn is used for the overshooting of temperature near the surface of rotating disk. Thermophoresis parameter *Nt* influence on temperature distribution $\theta$($\zeta$) is elucidated in Fig. 14. Heat transfer is decreased for larger values of *Nt*. The non-dimensional thermal relaxation time parameter $\gamma _{3}$ and temperature profile $\theta$($\zeta$) role is discussed through Fig. 15. Heat transfer is effectively enhanced via $\gamma _{3}$. It means that Cattaneo-Christov heat flux overcomes the heat capabality of the fluid. The scenerio is justified as when $\gamma _{3}$ is magnified, the Oldroyd-B nanofluid particles transfer more heat to nearby particles and hence temerature of the sytem is made high. Supplementary heat is generated by the ineraction of nanoparticles and the base fluid due to Cattaneo-Christov heat transfer. Consequently, the thermal boundary layer is made thicker and the influence is so prominent that enhanced conduction of heat is noted in the vicinity of the disk. If $\gamma _{3}$ = 0, Cattaneo-Christov heat transfer model becomes the classical Fourier’s law for heat transfer.

**Figure 16:**
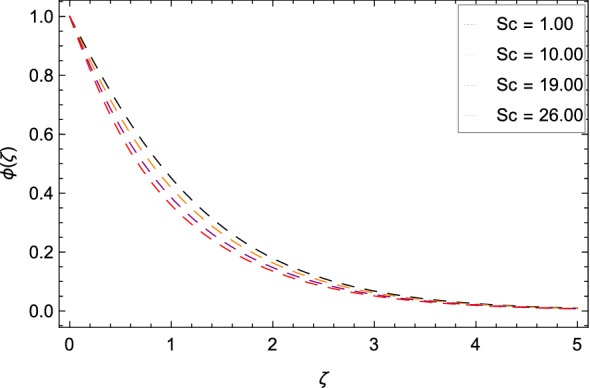
Pattern of graph curves on account of *Sc*.

**Figure 17:**
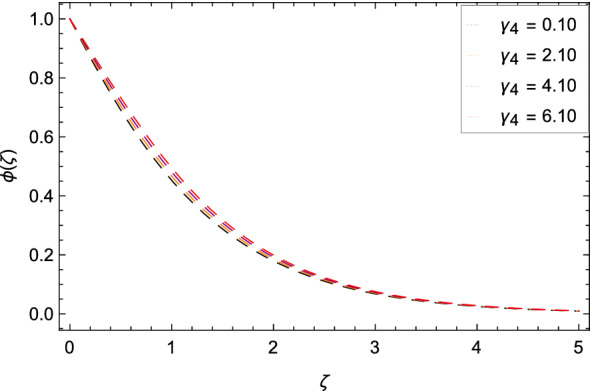
Pattern of graph curves on account of $\gamma _{4}$.

**Figure 18:**
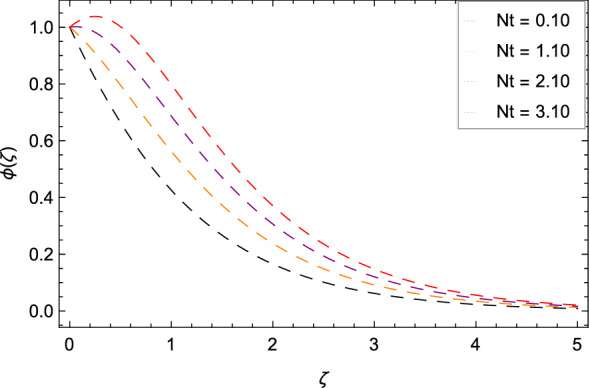
Pattern of graph curves on account of *Nt*.

**Figure 19:**
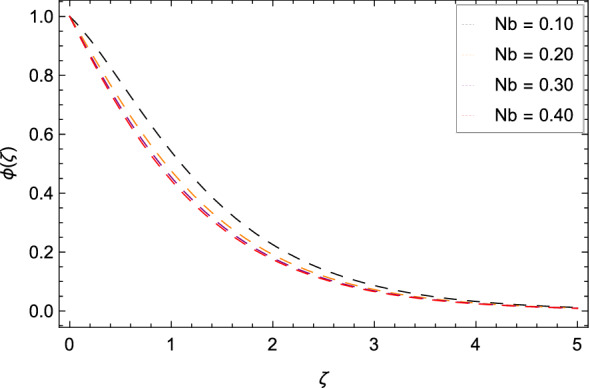
Pattern of graph curves on account of *Nb*.

Figure 16 interpretes that nanoparticles concentration $\phi$($\zeta$) is declined with the high estimations of Schmidt number *Sc*. The reason is that the amount change in concentration across the ambient and the surface is minimum. Figure 17 reports the performances of non-dimensional solutal relaxation time parameter $\gamma _{4}$ and nanoparticles concentration $\phi$($\zeta$). The concentration of nanoparticles is enhanced with the high values of $\gamma _{4}$. The observation shows that Cattaneo-Christov mass flux has better results. Figure 18 indicates that the thermophoresis parameter *Nt* increases the nanoparticles concentration $\phi$($\zeta$) profile. Physically, the addition of *Nt* maximizes the thermal gradient in the present system due to thermophoresis in which nanoparticles move away from a higher heating location to a lower location level which increases the concentration. Due to thermophoresis an additional heat is generated by the interation nanoparticles with other particles and base fluid which intensify the nanoparticles concentration $\phi$($\zeta$). Nanoparticles concentration $\phi$($\zeta$) for the Brownian motion parameter *Nb* is decreased as depicted by Fig. 19. The reason is that through sufficient large values of *Nb*, the nanoparticles diffusion is not remarkable. In Buongiorno nanofluid model, the parameter *Nb* is inversely proportional to the size of nanoparticles. So with high values of *Nb*, smaller nanoparticles are exist which decrease the nanoparticles concentration profile $\phi$($\zeta$) due to the random motion.

**Figure 20:**
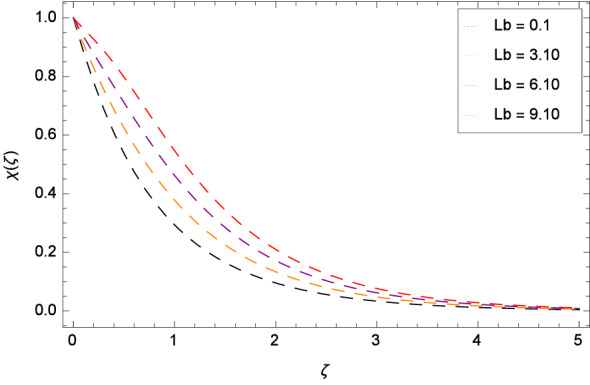
Pattern of graph curves on account of *Lb*.

**Figure 21:**
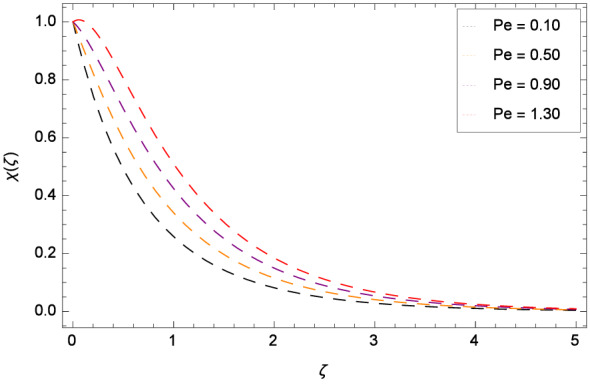
Pattern of graph curves on account of *Pe*.

**Figure 22:**
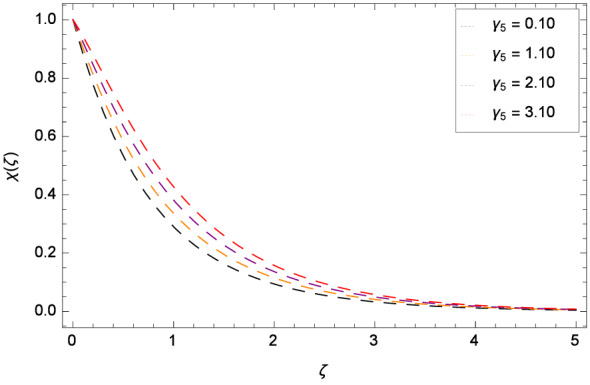
Pattern of graph curves on account of $\gamma _{5}$.

**Figure 23:**
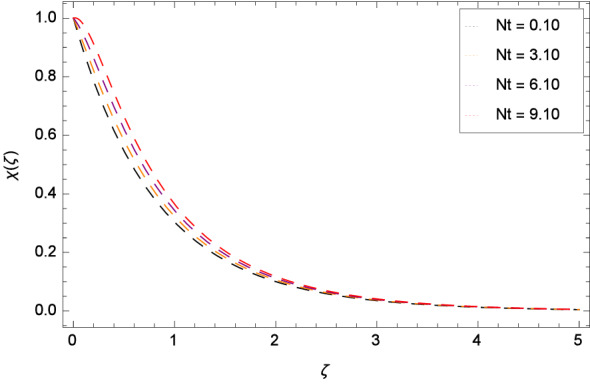
Pattern of graph curves on account of *Nt*.

**Figure 24:**
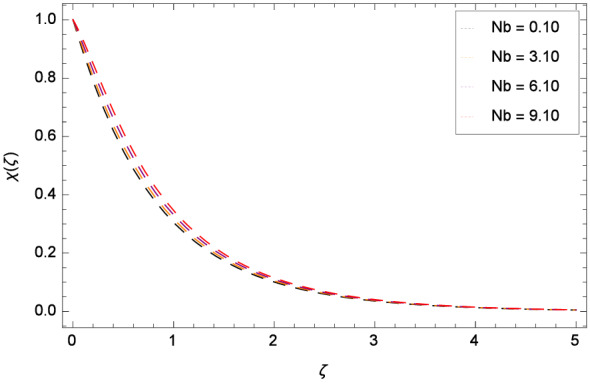
Pattern of graph curves on account of *Nb*.

The bioconvection Levis number *Lb* effect is shown through the Fig. 20. Bioconvection Levis number is generated on account of taking the ratio of diffusivities of momentum to mass of gyrotactic microorganisms. So due to the existence of these diffusivities, concentration $\chi$($\zeta$) of gyrotactic microorganisms is enhanced. It encourages the mixing and species diffusion in the nanofluid. Figure 21 is sketched to express the increment of gyrotactic microorganisms concentration $\chi$($\zeta$) with Peclet number *Pe*. The increment in gyrotactic microorganisms concentration promotes the diffusion into the boundary layer of nanoparticles and gyrotactic microorganisms. Outcome of the parameter $\gamma _{5}$ is exhibited by Fig. 22. Favorable role uplifts the $\chi$($\zeta$). Figure 23 exhibits that *Nt* amplifies the $\chi$($\zeta$) profile. Similarly, in Fig. 24, it is exhibited that $\chi$($\zeta$) boosts with *Nb* .

**Figure 25:**
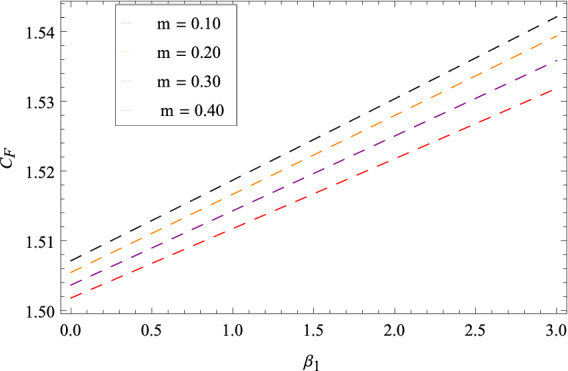
Pattern of graph curves on account of *m*.

**Figure 26:**
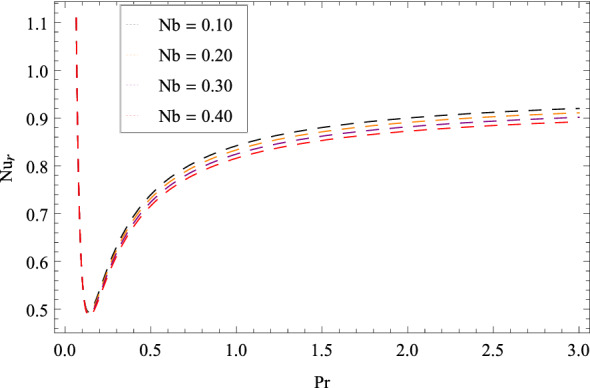
Pattern of graph curves on account of *Nb*.

**Figure 27:**
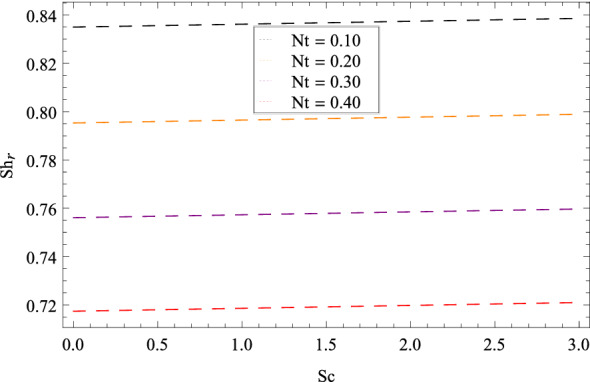
Pattern of graph curves on account of *Nt*.

**Figure 28:**
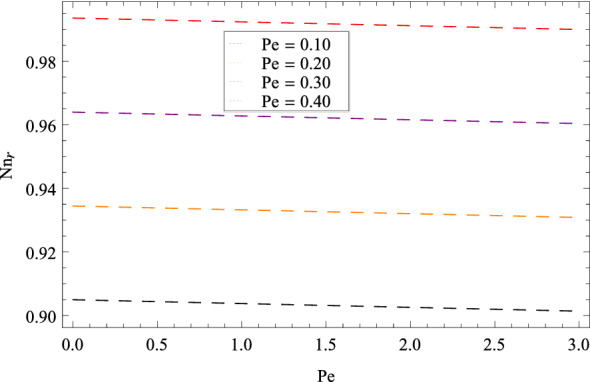
Pattern of graph curves on account of *Pe*.

Figure 25 shows that skin friction $C_{F}$ is decreased with the increasing values of Hall parameter *m*. Similarly in Fig. 26, it is revealed that the Nusselt number $Nu_{r}$ is a decreasing function of Brownian motion parameter *Nb*. The Sherwood number $Sh_{r}$ versus Schmidt number *Sc* is shown in Fig. 27. It is observed that the Sherwood number is weakened with enhancing values of thermophoresis parameter *Nt*. On contrary to the previous cases, the motile microorganisms number $Nn_{r}$ is growing large with the positive increasing values of Peclet number *Pe* as shown in Fig. 28. Tables 2, 3, 4 and 5 depict the tabulated data of skin friction coefficient, Nusselt number, Sherwood number and motile microorganisms number respectively.

**Table 2:** Numerical values of skin friction coefficient.

*m*	$\beta _{1}$	$\beta _{2}$	$\gamma _{3}$	$\gamma _{4}$	$\Omega _{1}$	$\lambda _{3}$	$C_{F}$
0.70	0.20	0.30	0.20	0.40	0.50	0.60	1.52082
1.70	–	–	–	–	–	–	1.51382
2.70	–	–	–	–	–	–	1.51478
–	1.20	–	–	–	–	–	1.54221
–	2.20	–	–	–	–	–	1.56430
–	–	1.30	–	–	–	–	1.36407
–	–	2.30	–	–	–	–	1.21920
–	–	–	1.20	–	–	–	1.21920
–	–	–	2.20	–	–	–	1.52082
–	–	–	–	1.40	–	–	1.52082
–	–	–	–	2.40	–	–	1.52082
–	–	–	–	–	1.50	–	3.37967
–	–	–	–	–	2.50	–	6.89973
–	–	–	–	–	–	1.60	1.71262
–	–	–	–	–	–	2.60	1.91410

**Table 3:** Numerical values of Nusselt number.

*m*	$\beta _{1}$	$\beta _{2}$	$\gamma _{3}$	$\gamma _{4}$	$\Omega _{1}$	$\lambda _{3}$	$Nu_{r}$
0.70	0.20	0.30	0.20	0.40	0.50	0.60	0.913289
1.70	–	–	–	–	–	–	0.903289
2.70	–	–	–	–	–	–	0.912289
–	1.20	–	–	–	–	–	0.913389
–	2.20	–	–	–	–	–	0.913489
–	–	1.30	–	–	–	–	0.913589
–	–	2.30	–	–	––	–	0.913689
–	–	–	1.20	–	–	–	0.913789
–	–	–	2.20	–	–	–	0.913889
–	–	–	–	1.40	–	–	0.913989
–	–	–	–	2.40	–	–	0.913289
–	–	–	–	–	1.50	–	0.914049
–	–	–	–	–	2.50	–	0.914809
–	–	–	–	–	–	1.60	0.913289
–	–	–	–	–	–	2.60	0.913289

**Table 4:** Numerical values of Sherwood number.

*m*	$\beta _{1}$	$\beta _{2}$	$\gamma _{3}$	$\gamma _{4}$	$\Omega _{1}$	$\lambda _{3}$	$Sh_{r}$
0.70	0.20	0.30	0.20	0.40	0.50	0.60	0.856733
1.70	–	–	–	–	–	–	0.806733
2.70	–	–	–	–	–	–	0.816733
–	1.20	–	–	–	–	–	0.826733
–	2.20	–	–	–	–	–	0.836733
–	–	1.30	–	–	–	–	0.846733
–	–	2.30	–	–	–	–	0.856733
–	–	–	1.20	–	–	–	0.866733
–	–	–	2.20	–	–	–	0.876733
–	–	–	–	1.40	–	–	0.886733
–	–	–	–	2.40	–	–	0.896733
–	–	–	–	–	1.50	–	0.856419
–	–	–	–	–	2.50	–	0.856104
–	–	–	–	–	–	1.60	0.856733
–	–	––	–	–	–	2.60	0.856733

**Table 5:** Numerical values of motile density number.

*m*	$\beta _{1}$	$\beta _{2}$	$\gamma _{3}$	$\gamma _{4}$	$\Omega _{1}$	$\lambda _{3}$	$Nn_{r}$
0.70	0.20	0.30	0.20	0.40	0.50	0.60	0.981355
1.70	–	–	–	–	–	–	0.980355
2.70	–	–	–	–	–	–	0.982355
–	1.20	–	–	–	–	–	0.983355
–	2.20	–	–	–	–	–	0.984355
–	–	1.30	–	–	–	–	0.985355
–	–	2.30	–	–	–	–	0.986355
–	–	–	1.20	–	–	–	0.987355
–	–	–	2.20	–	–	–	0.988355
–	–	–	–	1.40	–	–	0.989355
–	–	–	–	2.40	–	–	0.981355
–	–	–	–	–	1.50	–	0.980355
–	–	–	–	–	2.50	–	0.980355
–	–	–	–	–	–	1.60	0.981355
–	–	–	–	–	–	2.60	0.981355

## Conclusions

The present problem can modelled in double disk with different effects which will pave the ways for further investigations.

The main results are summarised as following.

1. The radial velocity is reduced with the increasing values of relaxation time, retardation time and magnetic field parameters while it is enhanced with porosity, Darcy Forchheimer and stretching parameters.
2. The azimuthal velocity is enhanced with the increasing values of relaxation time, retardation time, magnetic field, porosity and Darcy Forchheimer parameters.
3. The temperature is reduced with the increasing values of Brownian motion and thermophoresis parameters while it is enhanced with the thermal relaxation time parameter.
4. The nanoparticles concentration is reduced with the increasing values of Schmidt number and thermophoresis parameter while it is enhanced with solutal relaxation time and Brownian motion parameters.
5. The gyrotactic microorganisms concentration is enhanced with the increasing values of Peclet number, thermophoresis parameter, bioconvection Levis number, gyrotactic microorganisms concentration difference and Brownian motion parameters.
6. The comutational work has close agreement with published material.

## Data Availability

Upon reasonable request to the corresponding author, the data will be provided.
